# Sleep pattern and predictors of daily versus as-needed hypnotics use in middle-aged and older adults with insomnia

**DOI:** 10.1186/s12875-022-01707-w

**Published:** 2022-05-02

**Authors:** Maria Tanielian, Jumana Antoun, Munir Sidani, Ahmad Halabi, Malak Hoballah, Kegham Hawatian, Georges Assaf

**Affiliations:** 1 Department of Family Medicine, American University of Beirut Medical Center, Hamra, Beirut, Lebanon; 2 Faculty of Medicine, American University of Beirut Medical Center, Beirut, Lebanon; 3 Department of Academic Internal Medicine and Geriatrics, the University of Illinois at Chicago, Chicago, IL USA

## Abstract

**Introduction:**

This study aims to examine the sleep pattern and predictors of daily vs. as-needed use of hypnotics in middle-aged and older adults with insomnia.

**Methods:**

Patients aged 50–75 who use hypnotics for insomnia were identified via electronic medical records and were recruited. Data about sociodemographics, mood and cognitive screening measures, and questions related to sleep patterns were collected through an interview conducted over the phone.

**Results:**

A sample of 66 participants was recruited, of which 69.7% were females. Three quarters (49/66, 74.2%) used hypnotics daily, with 43% (21/49) of daily hypnotics users sleeping more than 8 h per night. Two-fifths (26/66, 39.4%) of participants still had clinically significant insomnia even after taking hypnotics. After adjusting for age, years of hypnotics use, sleeping hours per night, PHQ-2 score, and frequency of pain at night, the logistic regression model showed that younger age (*p* = 0.023) and longer sleeping hours per night (*p* = 0.025) were significantly associated with daily hypnotics use when compared to as needed hypnotics use.

**Conclusion:**

Many hypnotic users still have clinically significant insomnia and poor quality of sleep as reflected by perceived longer sleep duration and more daytime napping which could be related to drug-related residual sedation. Hypnotic use may not be the best solution for insomnia treatment in an older population, and physicians should regularly reassess the use of hypnotics.

## Introduction

Insomnia symptoms are common in older adults. These symptoms are termed sleep disturbance when they result into daytime consequences or patient dissatisfaction, impacting the quality of life [1]. Insomnia disorder is diagnosed when insomnia symptoms of a patient with sleep disturbance meet the Diagnostic and Statistical Manual of Mental Disorders Criteria of Insomnia (Frequency of symptoms: 3 or more nights/week, Duration of symptoms: at least 3 months, associated with dissatisfaction with sleep and daytime consequences) [1, 2].

Estimates of the prevalence of insomnia are variable in the literature because of inconsistency in definitions used and limitations in accurately estimating incidence and remission rate of insomnia [3]. The prevalence of insomnia, in older adults, is estimated to be between 13 and 47% [3]. The high prevalence of insomnia in older adults can be explained by sleep pattern changes that occur with age. As people age, their total sleep time (TST) decreases [4, 5], their sleep efficiency worsens, and they wake up more frequently than younger people [4, 6, 7]. Due to physiological changes that occur with age, older adults require less sleep time at night than younger adults. According to the National Sleep Foundation’s sleep time duration recommendations, sleeping 7–9 h per night for adults and 7–8 h per night for older adults is appropriate [8]. It has been shown that both short or long sleep duration (≤6 or ≥ 9 h per night) could have negative health outcome [9–11]. In a systematic review with meta-analysis sleep duration (both short and long) was associated with all-cause mortality [12].

Treating insomnia in older adults is a clinical challenge. Before starting treatment for insomnia, it is crucial to identify clinical insomnia from typical age-related sleep pattern changes [13]. If clinical insomnia is detected, it is important to rule out secondary causes of insomnia such as psychiatric illness or other medical conditions and treat accordingly. For primary clinical insomnia, non-pharmacologic treatments are recommended to be tried first [14–16]. For example, multicomponent cognitive-behavioral therapy for insomnia (CBTi) is effective when delivered in primary care and has successfully treated insomnia in older adults [14–17]. Although hypnotic use has been reported frequently among community-dwelling older adults, it is not recommended in this age group [18, 19]. Older patients have multiple chronic conditions and are at increased risk of polypharmacy. Polypharmacy is associated with an increased risk of inappropriate prescribing and adverse drug events [18–20]. Beers criteria, published by the American Geriatrics Society, recommends that older adults avoid hypnotics, including benzodiazepines and non-benzodiazepine gamma-aminobutyric acid agonists sometimes referred to as “Z-Drugs “due to their association with increased risk of delirium, falls, fractures, and cognitive impairment [19].

Few articles in the literature compare the effect of hypnotic use on sleep patterns compared to placebo in older adults [20–22]. A meta-analysis of 24 studies of hypnotic use in older adults (2417 participants) showed that the magnitude of the effect of improvement in sleep is small [20], and its use is associated with adverse cognitive and psychomotor events and daytime fatigue compared to placebo [20]. Sleep quality improvement (effect size 0.14), total sleep time increase (a mean of 25.2 min), and decrease in the number of nighttime awakenings (0.63) were significant in hypnotic users compared to placebo but with a small effect size. According to the same meta-analysis, 13 people need to be treated with a hypnotic drug for only one person to benefit, whereas six people need to be treated for one person to be harmed, indicating that the risk of harm is more than twice as likely as benefit [20, 21]..

Although several predictors of chronic hypnotic use have been described, including alcohol consumption, smoking, sleep difficulties, old age, female gender, and comorbid psychiatric disorders [23, 24], no articles to our knowledge studied the predictors of daily vs. as-needed use of hypnotics on sleep patterns. However since age [1–3], depressed mood [25, 26], and pain [25] are highly associated with insomnia in the literature, it could also be possible that these factors might predict the frequency of use of hypnotics for insomnia (Daily vs As needed). In addition length of time the hypnotic pill used (extent of its chronicity) and the length of sleeping time at night (Effected by hypnotic use) [27] might also predict the frequency of hypnotic use (Daily vs as needed).

This cross-sectional study aims to examine the sleep patterns of middle-aged and older adults with insomnia who use hypnotics and to study whether a set of factors (Age, pain, mood, sleep duration, chronicity of hypnotic use) predict daily hypnotic use compared to an as-needed basis use. Understanding the sleep patterns of older adults who use hypnotics for insomnia and understanding the predictors of frequency of hypnotic use (daily vs. as needed) will help physicians better address the sleep problems of older adults who use hypnotics with behavioral interventions. This knowledge could help them in tapering and eventually stopping hypnotic use.

## Methods

### Study population and design

The study has a cross-sectional design and was approved by the American University of Beirut Institutional Review Board (IRB). Electronic medical records were used to generate the names and contact information of patients who met the inclusion criteria. Patients aged 50 to 75 who visited the American University of Beirut Medical Center (AUBMC) Family Medicine Clinics between September 1, 2018, and May 1, 2021, and who were taking hypnotics for insomnia were included in the study. Hypnotics use for insomnia was defined as any of the following medications for insomnia: Benzodiazepines, non-benzodiazepine gamma-aminobutyric acid agonists, flupentixol, and melitracen combined, doxepin, amitriptyline, imipramine, desipramine, mirtazapine, diphenhydramine, doxylamine, and hydroxyzine. Exclusion criteria included: patients younger than 50 years old or older than 75 years old, patients who were not taking hypnotics or taking hypnotics for reasons other than insomnia, impaired decisional capacity to give consent or understand required questions, and severe hearing loss. After being contacted and screened for inclusion and exclusion criteria, eligible patients were invited to participate in the research study. Oral consent was obtained. Trained researchers collected sociodemographic data, mood and cognitive screening measures, and questions about their sleep patterns.

Since the study involves less than minimal risk, an explicit assessment of decisional capacity was not assessed. However, the patient’s decisional capacity to consent to participate in the study was measured by a 2 step process. The first step involves a quick determination of the need for a detailed assessment —the subject was asked: “Can you tell me what this study is about?” An adequate answer to this question may eliminate the necessity for further evaluation of the decisional capacity. If the patient fails to give an acceptable answer, the research assistant will proceed to step two and the subject will be asked to answer the University of California Brief Assessment of Capacity to Consent (UBACC) which is a 10-item questionnaire with a maximum score of 20 [28] (13). A score of ≤12 disqualifies the patient from participating in the study. Once the patient is determined to have the decisional capacity and consents to participate in the study, a researcher asked them the research questions.

### Sleep pattern measures

Insomnia severity was determined using the Insomnia Severity Index (ISI) [29, 30]. ISI is a validated, reliable tool for evaluating perceived sleep difficulties in the general population with adequate internal consistency. It has a 7 question inventory with a one-month recall period. A 5 point scale is used to rate each question. The score ranges from 0 to 28. The interpretation of the score is as follows: 0–7 no clinically significant insomnia, 8–14 subthreshold insomnia, 15–21 moderate insomnia, 22–28 severe insomnia. The first 3 questions of ISI, which include difficulty falling asleep, difficulty staying asleep and the problem with waking up too early are interpreted separately in addition to the global ISI score.

Chronotype and other detailed sleep parameters were determined by the Munich Chronotype Questionnaire (MCTQ) [31, 32]. MCTQ is a questionnaire that measures chronotype. Chronotype is the natural inclination of one’s body to sleep at a particular time. Some older adults have an inclination to go to sleep early at night (These are called M-types or morning types), others have an inclination to go to sleep late at night (These are called E-types or evening types). MCTQ consists of several questions about the bed and sleep-related time separately on workdays and off days. MSFSc (Mid-sleep on off days corrected for sleep debt on workdays) was used to measure working older adults’ chronotype, while MSF (Mid-sleep on off days) was used for chronotype measurement in non-working participants. Both of these measures are included in MCTQ. MSFsc or MSF scores below 2.17 are classified as extreme M-types (Morning types), and MSFsc or MSF scores above 7.25 were classified as extreme E-types (Evening types).

Mid sleep is Midpoint between sleep onset and wake time. MSF is calculated by the following formula: MSF = (Sleep onset + sleep duration)/2. MSFsc is calculated by the following formula: MSFsc = MSF- (Sleep duration on free days-average weekly sleep duration)/2.

### Depression and cognitive screening measures

Patient Health Questionnaire 2 (PHQ-2), a short 2 item questionnaire, was used to screen for depression [33]. The two questions are each on a 4 point Likert scale. PHQ-2 scores range from 0 to 6. If the score was three or greater, major depressive disorder was considered likely, and the patient was asked to fill the Patient Health Questionnaire-9 (PHQ-9). PHQ-9 is a 9 item questionnaire with a 4 point Likert scale for each item. Total scores of 5, 10, 15, and 20 represent cut points for mild, moderate, moderately severe, and severe depression, respectively [34]. Participants who have met the criteria for moderate or severe depression by PHQ-9 and were not already following up for depression were informed about the results and instructed to follow up with their primary care physician for further investigation and treatment.

Patients were asked to repeat five words (table, sky, cat, table, car) and were instructed to remember them and recall the five words 5 min. later. The 5-word recall test is a short and simple screening test to assess for episodic memory. The more words patients were able to recall, the better their episodic memory was considered [35]. Depression assessment was done (PHQ-2 questionnaire) between encoding and recall.

The ISI, PHQ-2, and PHQ-9 were previously translated to Arabic and validated in Arabic-speaking populations in the literature [36–38]. The MCTQ was translated to Arabic by a certified translator and was content-validated by an expert committee of sleep specialists at AUBMC.

#### Pain and hypnotic use measures

Pain frequency and intensity were measured as follows: We asked the participants whether they had pain at night (yes/no) and if yes, how frequent is it per week and how intense is it. Pain frequency/week choices were measured on a scale from 1 to 3. 1.- no pain 2.- pain only few days per week, 3.- pain half days per week or more. For pain intensity, participants were asked to give a scale to their pain from 0 to 10.

Hypnotic use coded as daily use or non-daily use. The patients were asked if they have previously tried to stop the pill and the answer is documented as yes or no. The patients were also asked if they had a current wish to stop the pill and similarly the answer is documented as yes or no.

### Statistical analysis

Descriptive statistics included frequencies and proportions for categorical data and means with standard deviations (SD) for continuous data. Pearson correlation was performed to find the association between ISI and PHQ-2 and PHQ-9. Logistic regression analysis was performed to explore whether a set of five factors may predict daily hypnotic users compared to as-needed hypnotic users. The dependent variable was daily hypnotic use vs. as-needed hypnotic use. The independent variables were Age, PHQ-2 score, pain at night, duration of hypnotics used, and sleep duration. SPSS statistical package (SPSS Inc., Chicago, IL, USA, version 27) was used for data analysis. Using G*Power 3.1.9.7, a sample size of 68 was needed for 5 predictors and effect size measured by Cohen’s f2 of 0.15 with an alpha level of 0.05 and power of 80%.

## Results

### General characteristics of participants

A sample of 66 participants was recruited, of which 69.7% (46/66) were females. The mean age of participants was 63.5 years, and 74.2% (49/66) were unemployed. Table 1 shows the baseline characteristics of the participants. Daily use of hypnotics was reported among three-quarters of the participants (49/66, 74.2%), while the rest (17, 25.8%) used hypnotics as needed during the week (At least once per week). More than half (34/66, 51.5%) have previously tried to stop the hypnotic, and one-third (20/66, 30.3%) have present wishes to do so. Benzodiazepines were the most often used hypnotics for sleep (48/66, 72.7%). Non-benzodiazepine gamma-aminobutyric acid agonist users accounted for 27 (40.9%) of participants. Other drugs used by the remaining 11 (16.6%) participants included tricyclic antidepressants (TCAs), quetiapine, and flupentixol and melitracen combined.

**Table 1:** General characteristics of participants (*n* = 66)

Variables	Number (%)
**Age**	
50–65	39 (59.1%)
66–75	24 (36.4%)
**Gender**	
Male	20 (30.3%)
Female	46 (69.7%)
**Marital status**	
Married	41 (62.1%)
Single	6 (9.1%)
Divorced	4 (6.1%)
Widowed	15 (22.7%)
**Education**	
Below high school	17 (25.8%)
High school	21 (31.8%)
College	9 (13.6%)
Bachelor	18 (27.3%)
**Employed**	
Yes	17 (25.8%)
No	49 (74.2%)
**Daily use of caffeinated drinks**	
yes	64 (97%)
No	2 (3%)
**Alcohol use**	
Less than weekly	59 (89.4%)
Weekly or more	6 (9.1%)
**Smoking**	
yes	40 (60.6%)
no	26 (39.4%)
**Frequency of hypnotics use**	
Daily	49 (74.2%)
As needed	17 (25.8%)
**Duration of hypnotics use**	
< 1	4 (6.1%)
1–5	29 (43.9%)
5.1–10	12 (18.2%)
> 10	16 (24.2%)
**Previous trial to stop hypnotics**	
Yes	34 (51.5%)
No	32 (48.5%)
**Current wish to stop hypnotics**	
Yes	20 (30.3%)
No	46 (69.7%)
**PHQ-2 score**	
2 or less	36 (54.5%)
3 or more	30 (45.5%)
**Pain at night**	
None	34 (51.5%)
Some days (Less than half of days per week)	10 (15.2%)
Half of days per week or more	22 (33.3%)
**Financial difficulty**	
None	16 (24.2%)
Mild	12 (18.2%)
Moderate	24 (36.4%)
Severe	14 (21.2%)

Of all participants, 26 (39.4%) still had clinically significant insomnia even after taking hypnotics, of which 15 (22.7%) had subthreshold insomnia, 9 (13.6%) had moderate severity clinical insomnia, and 2 (3%) had severe clinical insomnia. Two-thirds (43/66, 65%) reported they take daily naps. Around one third of participants (21/66, 31.8%) reported they need more than 30 min to fall sleep. Two-thirds (46/66, 69.7%) reported they wake up at least once in the middle of the night. One-third (23/66, 34.8%) wake up more than two times in the middle of the night. When the participants were asked how much time in general they require to fall sleep once they wake up in the middle of the night during each awakening, One third (21/66, 31.8%) of participants did not answer the question. Three quarter of those who answered the question (36/45, 80%) indicated they need less than 30 min to fall back to sleep and one quarter (9/45, 20%) indicated they need more than 30 min to fall back to sleep. The most common sleep complaint was trouble staying asleep by 27 participants (40.9%), followed by trouble falling asleep by 19 participants (28.78%) and early morning awakening by 11 participants (16.6%). One-third (22/66, 33%) slept more than 8 h per night. The mean extra sleeping time in those who slept more than 8 h per night was 84 min (Range: 15–398 extra minutes above the 8 h maximum recommended sleep time).

There was no association between the insomnia severity (ISI) and the various demographics except for depression. There was a positive correlation between ISI and PHQ-2 (Pearson correlation 0.294, *P* = 0.017) and a positive correlation between ISI and PHQ-9 (Pearson correlation 0.624, *P* < 0.0001).

Sleep-related parameters stratified by frequency of hypnotics use (daily vs. as-needed) are presented in Table 2.

**Table 2:** Sleep-related parameters stratified by frequency of hypnotics use (daily vs. as-needed)

	Daily use of hypnotics ***N*** = 49 (%)	As-needed use of hypnotics ***N*** = 17 (%)	Total N = 66 (%)
**Age**			
50–65	33 (67.3%)	6 (35.3%)	39 (59%)
66–75	16 (32.7%)	11 (64.7%)	27 (41%)
**Gender**			
Male	15 (30.6%)	5 (29.4%)	20 (30%)
Female	34 (69.4%)	12 (70.6%)	46 (70%)
**Pain at night**			
No pain	24 (49%)	10 (58.8%)	34 (51.5%)
Few days per week	5 (10.2%)	5 (29.4%)	10 (15.2%)
Half days per week or more	20 (40.8%)	2 (11.8%)	22 (33.3%)
**Sleep duration (hours)**			
Up to 8	28 (57%)	16 (94%)	44 (66.7%)
More than 8	21 (43%)	1 (6%)	22 (33.3%)
**Time to fall sleep**			
Up to 30 min	35 (71.4%)	10 (58.8%)	45 (68.2%)
More than 30 min	14 (28.6%)	7 (41.2%)	21 (31.8%)
**Waking up in middle of night**			
No	17 (34%)	3 (17.6%)	20 (30.3%)
Yes	32 (64%)	14 (82.4%)	46 (69.7%)
**Approximate time to fall asleep after each awakening in the middle of the night**^**a**^			
Up to 30 min	27 (81.8%)	9 (75%)	36 (80%)
More than 30 min	6 (18.2%)	3 (25%)	9 (20%)
**Daily Naps**			
yes	33 (67.3%)	10 (58.8%)	43 (65.1%)
No	16 (32.7%)	7 (41.2%)	23 (34.9%)
**Duration of hypnotics used (in years)**^**a**^			
0–10	30 (67%)	15 (94%)	45 (73.7%)
Above 10	15 (33%)	1 (6%)	16 (26.3%)
**PHQ-2 score**			
0–2	25 (51%)	11 (64.7%)	36 (54.5%)
3 and above	24 (49%)	6 (35.3%)	30 (45.5%)
**Chronotype**			
Less than 2.17	7 (14.3%)	2 (11.8%)	9 (13.6%)
2.17–7.25	42 (85.7%)	15 (88.2%)	57 (86.4%)
**Insomnia severity index (ISI)**			
0–7	30 (61.2%)	10 (58.8%)	40 (60.6%)
8–28	19 (38.8%)	7 (41.2%)	26 (39.4%)

^a^There are 21 missing values in this sleep parameter. Also to note this does not represent the WASO (Total wakefulness time after sleep onset). This represents the maximum time someone stays awake after each waking up in the middle of the night

### Predictors of daily versus as-needed hypnotics use

Logistic regression was performed to predict if the above-mentioned five independent variables (Age, PHQ-2, pain at night, duration of hypnotics used, and sleep duration) predict the daily use of hypnotics use compared to as-needed use of hypnotics. The logistic regression model was statistically significant, *X* [2] (5) = 24.648, *p* < 0.0001. The model explained 48.6% (Nagelkerke R [2]) of the variance in daily vs as-needed hypnotic use.

Multivariate analysis model revealed that relatively younger age and longer sleeping hours per night significantly predicted daily use of hypnotics compared to as-needed use of hypnotics (Table 3). A secondary analysis was performed by adding two additional variables in the above mentioned logistic regression model. The two added continuous variables were “time it takes to fall sleep” and “number of waking up in the middle of the night”. The model with 7 variables was analyzed and remained statistically significant, *X* [2] (5) = 26.235, *p* < 0.0001. In this model, still younger age (*P* value = 0.024) and longer sleeping hours per night (*P* value = 0.023) remained the only two significant predictors of daily use of hypnotics compared to as-needed use of hypnotics.

**Table 3:** Multivariate analysis of factors associated with frequency of hypnotics use (daily vs. as-needed use)***

Variables	Odds Ratio	95% CI	***P***-Value
**Age**	0.866	0.764–0.980	0.023
**Duration of hypnotics used**	1.134	0.988–1.302	0.074
**Pain at night**^**a**^	1.370	0.901–2.081	0.141
**Sleep duration**^**b**^	1.010	1.001–1.018	0.025
**PHQ-2**	0.849	0.563–1.281	0.436

^a^Pain at night is a scale that ranges from 0 to 5, where 0 is no pain on any day during the week, and 5 is pain every day during the week

^b^Sleep duration is the time between when the patient falls to sleep at night and wakes up in the morning; it is a continuous variable measured in minutes

***The significance of this model is (*P* < 0.0001)

## Discussion

We found that around 40% of the study sample had clinically significant insomnia, as measured by ISI score, despite hypnotics use. Considering that as-needed users of the hypnotic were asked to answer the ISI questions based only on the nights when they take the hypnotic pills, high ISI scores indicate residual insomnia despite using the hypnotic pill. The high prevalence of insomnia in this sample of older adults who use hypnotics (either daily or as needed) highlights the lack of effectiveness of hypnotics as a treatment for insomnia in older adults. These results are consistent with previous studies showing that prescription hypnotics are ineffective in treating insomnia in older adults over the long term [39]. Sleep maintenance was found to be the most common sleep complaint in our study sample in concordance with the literature [40]. In a randomized controlled trial comparing sleep maintenance between older persons (50–60 years old) and young adults (20–30 years old) using an electroencephalograph, older adults wake up more frequently at night than their younger counterparts [41].

The results of the logistic regression showed that participants who took the hypnotics daily slept more hours at night than participants who took the hypnotics on an as-needed basis. In addition, 43% of those who used hypnotics daily slept more than 8 h per night, which means they slept more than the daily sleep time duration hour recommended for their age group according to the National Sleep Foundation’s sleep time duration recommendations. Despite complaining of insomnia and taking hypnotic pills, sleeping more than the recommended sleep hours for age could be attributed to residual sedation from hypnotics or inadequate sleep. Approximately three-quarters of our study population used benzodiazepine hypnotic, which is known to be associated with daytime sedative effects, excessive sedation [27], and deep sleep suppression, compromising sleep’s restorative effects [42]. Furthermore, frequent naps throughout the day were reported by 65% of the participants. Daytime naps could contribute to self-perpetuating nighttime insomnia [43] and subsequent hypnotics use. This vicious cycle in which disturbed sleep leads to daytime napping and disturbed nighttime sleep may be improved by behavioral therapies, such as sleep hygiene counseling.

Younger age was also found to be associated with an increased risk of daily hypnotic use. In contrast to the literature, advanced age has been associated with long-term hypnotics use [17, 23]. Most of the studies exploring hypnotics use in older patients with insomnia were conducted in high-income countries [22]. While there has been an increased use of long-term hypnotics during the 21st century among all age groups, psychotropic drug use varies according to the socio-cultural context and the regulatory environment [44–46]. In our sample, we hypothesize that the younger age group may have a more “liberal and relaxed” attitude towards daily hypnotics. As studies have shown that there is a stigma surrounding the use of sleeping pills and the older age group may have moral objections towards their use [47, 48]. Given the changing regulatory environment, access to hypnotics may now be more feasible for younger patients, which may not have been the case for older patients. The younger age group is more likely to continue long-term hypnotics use into older age as studies have shown that more than half of chronic insomnia patients who are started on hypnotics are likely to continue taking them long-term [49].

Approximately half of our sample have attempted but failed to stop taking hypnotics in the past, and 30.3% have expressed a current desire to stop. Our findings were concordant with previous studies showing that gradual taper of hypnotics alone is less likely to be successful [50]. Multicomponent Cognitive Behavioral Therapy for Insomnia (CBTi) is considered the first-line therapy for patients with chronic insomnia and was shown to be effective in older adults [51]. A recent meta-analysis of eight randomized controlled trials that all investigated CBT-I found that short-term (less than 3 months) CBT-I combined with gradual tapering was more effective than gradual tapering alone for ceasing hypnotics and reducing insomnia symptoms [52, 53]. Helping patients discontinue their hypnotics can be done by specialists, primary care physicians, and trained nurses. For instance, a hypnotic withdrawal program led by primary care nurses had been successful in helping older adults stop hypnotics use, with no associated reduction in sleep quality after discontinuation [54].

There were several limitations to this study. First, it has a cross-sectional design and consequently, we are unable to infer causal relationships. The study was conducted at a family medicine department at a single tertiary care academic center with a possible sampling bias. The sample is relatively small which limits generalizability. A larger sample size would have made it feasible to assess more variables that could potentially affect our outcome. The data on sleep patterns and hypnotics was self-reported and did not include drug dosages used by patients for insomnia which may have impacted our findings. In addition, medical comorbidities of patients which might affect sleep, including sleep apnea, were not addressed in this research. Future replication studies using objective measures such as actigraphy or polysomnographic study to examine sleep patterns and reviewing medical records for drug dosages and duration are important to further examine the effect of hypnotics use on sleep patterns and quality of sleep in older patients with insomnia.

## Conclusion

Many hypnotic users still have clinically significant insomnia and poor quality of sleep as reflected by perceived longer sleep duration and more daytime napping which could be related to drug-related residual sedation. Hypnotic use may not be the best solution for insomnia treatment in an older population, and physicians should regularly reassess the use of hypnotics.

## Data Availability

The datasets generated and/or analysed during the current study are available in the open science framework repository (https://osf.io/). https://mfr.osf.io/render?url=https%3A%2F%2Fosf.io%2Fhrnqu%2Fdownload

## Abbreviations

AUBMC: American University of Beirut Medical Center
CBTi: Cognitive-Behavioral Therapy for insomnia
ISI: Insomnia Severity Index
MCTQ: Munich Chronotype Questionnaire
MSF: Mid-sleep on off days
MSFSc: Mid-sleep on off days corrected for sleep debt on workdays
PHQ-2: Patient Health Questionnaire 2
PHQ-9: Patient Health Questionnaire-9
SD: Standard Deviations
TST: Total Sleep Time
